# A Signature of Circulating microRNAs Predicts the Susceptibility of Acute Mountain Sickness

**DOI:** 10.3389/fphys.2017.00055

**Published:** 2017-02-08

**Authors:** Bao Liu, He Huang, Gang Wu, Gang Xu, Bing-Da Sun, Er-Long Zhang, Jian Chen, Yu-Qi Gao

**Affiliations:** ^1^Institute of Medicine and Hygienic Equipment for High Altitude Region, College of High Altitude Military Medicine, Third Military Medical UniversityChongqing, China; ^2^Key Laboratory of High Altitude Environmental Medicine, Third Military Medical University, Ministry of EducationChongqing, China; ^3^Key Laboratory of High Altitude Medicine, PLAChongqing, China

**Keywords:** circulating microRNAs, signature, prediction, acute mountain sickness

## Abstract

**Background:** Acute mountain sickness (AMS) is a common disabling condition in individuals experiencing high altitudes, which may progress to life-threatening high altitude cerebral edema. Today, no established biomarkers are available for prediction the susceptibility of AMS. MicroRNAs emerge as promising sensitive and specific biomarkers for a variety of diseases. Thus, we sought to identify circulating microRNAs suitable for prediction the susceptible of AMS before exposure to high altitude.

**Methods:** We enrolled 109 healthy man adults and collected blood samples before their exposure to high altitude. Then we took them to an elevation of 3648 m for 5 days. Circulating microRNAs expression was measured by microarray and quantitative reverse-transcription polymerase chain reaction (qRT-PCR). AMS was defined as Lake Louise score ≥3 and headache using Lake Louise Acute Mountain Sickness Scoring System.

**Results:** A total of 31 microRNAs were differentially expressed between AMS and Non-AMS groups, 15 up-regulated and 16 down-regulated. Up-regulation of miR-369-3p, miR-449b-3p, miR-136-3p, and miR-4791 in patients with AMS compared with Non-AMS individuals were quantitatively confirmed using qRT-PCR (all, *P* < 0.001). With multiple logistic regression analysis, a unique signature encompassing miR-369-3p, miR-449b-3p, and miR-136-3p discriminate AMS from Non-AMS (area under the curve 0.986, 95%CI 0.970–1.000, *P* < 0.001, LR+: 14.21, LR–: 0.08). This signature yielded a 92.68% sensitivity and a 93.48% specificity for AMS vs. Non-AMS.

**Conclusion:** The study here, for the first time, describes a signature of three circulating microRNAs as a robust biomarker to predict the susceptibility of AMS before exposure to high altitude.

## Introduction

There are three main highland regions of the world that support large populations, including the Tibetan plateau and Himalayan valleys, the Andes of South America, and the Ethiopian highlands. With the development of economy and tourism, the number of individuals who ascended to high altitude for a variety of reasons is increasing. Acute mountain sickness (AMS) is a public health problem of persons who acutely ascended to high altitude with an elevation more than 2500 m (Hackett et al., 1976; MacInnis et al., 2013; Waeber et al., 2015). It may occur as early as 6–24 h following ascent (Wright et al., 2008). The primary clinical manifestation of AMS is a combination of several symptoms, such as headache, anorexia, dizziness, malaise, nausea, and sleep disturbance (Bärtsch and Swenson, 2013). Among these symptoms, headache is the cardinal symptom of AMS and is essential to diagnose AMS according to Lake Louise Scoring System (LLS; Roach et al., 1993). LLS is a simple and widely accepted tool for AMS assessment (Maggiorini et al., 1998), which was created at the Hypoxia Symposium in 1991 and later modified at the following Hypoxia Symposium in 1993. The LLS encompasses two parts, a self-report questionnaire including five symptoms (headache, gastrointestinal symptoms, dizziness, and difficulty sleeping) and a clinical assessment comprising three physical examination findings (altered mental status, ataxia, and peripheral edema). Severe AMS could make persons to be incapacitating during the period of exposure to high altitude and, more seriously, developed to high altitude cerebral edema which is a life-threatening form of acute altitude illness (Basnyat and Murdoch, 2003).

Given the high incidence of AMS among travelers and its deleterious effect on health, identifying those susceptible to the disorder would be useful as it would assist in the development of preventive strategies for a given individual. By far several prediction tools have been proposed, including cold pressor test (Kovtun and Voevoda, 2013), heart rate variability (Koehle et al., 2010; Karinen et al., 2012), and lung functions (Zhou et al., 2004). All these tools detected the physiological parameters of body, which were convenient and have low cost. Recently, there was several studies focus on gene features in the screening susceptibility of AMS (Ding et al., 2011). Although, the parameters measured by those above tools present significant association with the risk of AMS. The low sensitivity and specificity limited the use of those simple sea level tests (Song et al., 2013) and gene polymorphism detection method (Ding et al., 2011).

MicroRNAs are small (~22-nt long), noncoding, single-stranded RNAs that regulate gene expression post-transcriptionally in various physiological and pathophysiological cellular processes such as proliferation (Lenkala et al., 2014), differentiation (Shivdasani, 2006), metabolism (Dumortier et al., 2013), and apoptosis (Su et al., 2015). In recent years, researchers have found that microRNAs are also present in different body fluids, the one in blood refer to circulating microRNA (Weber et al., 2010). Circulating microRNAs are remarkably stable and can be specific to certain physiological and pathological conditions and are accessible to analysis through relatively non-invasive methods (Mitchell et al., 2008). The significant differences in their expression have been described in a wide array of various diseases, although their physiological functions remained unknown (Ai et al., 2010; Moussay et al., 2011; Roth et al., 2011).

Therefore, in the present study, we performed miRCURYTM LNA Array (v.18.0) (Exiqon) screening, followed by data validation with quantitative reverse-transcription polymerase chain reaction (qRT-PCR) to evaluate plasma microRNA profiles of individuals before exposure to high altitude to predict the susceptibility of AMS. Interestingly, our data indicated that a signature combined with circulating microRNAs miR-369-3p (MIMAT0000721), miR-449b-3p (MIMAT0009203), and miR-136-3p (MIMAT0004606) provided substantial AMS prediction.

## Methods and materials

### Participants

This study protocol was approved by the Ethics Committee of third Military Medical University, China, in accordance with the Declaration of Helsinki, and all individuals provided written informed consent before entry. Excluding individuals with a history of smoking and high altitude travel, 109 adult health volunteers aged 17–35 years whose primary residence was at an elevation of 1000 m or lower. Among them, 22 individuals were randomly selected for microRNA array screening, while the remaining 87 constituted the validation set with qRT-PCR method.

### Ascent and blood samples collection

All participants were driven from Chongqing (elevation, 200 m), China, before ascent. After a 3 day rest, they acutely ascended to Lhasa (elevation, 3648 m), China, by train within 48 h, where they remained for 5 days. Blood samples were collected in EDTA tubes using standard operating procedures in the morning before departure.

During the investigation, all volunteers had the same diet and were required to abstain from strenuous activity to ensure a similar level of physical activity. The investigators continuously monitored the individuals for evidence of high altitude pulmonary edema or high altitude cerebral edema according to the specific clinical manifestations of these diseases (Hackett and Roach, 2004; Pennardt, 2013). Immediate evacuation and treatment with oxygen were available for such an occurrence.

### Measurements

We measured main parameters, including blood pressure, heart rate, and blood oxygen saturation, prior to departure and successive 5 days after exposure to high altitude. AMS was diagnosed using the LLS. Self-report questionnaire of LLS were recorded prior to departure and at each day during high altitude exposure (5 days). Meanwhile, the clinical assessment was done by our accompanied doctors. As per the Lake Louise Consensus Group, individuals with a score of three points or greater on the AMS self-report questionnaire alone (including a headache score ≥ 1), or in combination with the clinical assessment score designated as AMS.

### RNA isolation and microRNA array analysis

According to manufacturers' instructions, total RNA was isolated using TRIzol (Invitrogen) and purified with RNeasy mini kit (QIAGEN). RNA quality and quantity were measured on a NanoDrop spectrophotometer (ND-1000, Nanodrop Technologies) and listed in Supplementary Table 1. After quality control, the miRCURY™ Hy3™/Hy5™ Power labeling kit (Exiqon, Vedbaek, Denmark) was used according to the manufacturer's guideline for microRNA labeling. After hybridized Hy3™-labeled samples on a miRCURYTM LNA Array (v.18.0) (Exiqon) and washed slides several times with a Wash buffer kit (Exiqon), the slides were scanned on an Axon GenePix 4000B microarray scanner (Axon Instruments, Foster City, CA). Scanned images were then imported into the GenePix Pro 6.0 software (Axon) for grid alignment and data extraction. Replicated microRNAs were averaged, and probes with intensities ≥30 in all samples were selected for normalization. Detailed instructions were as previously described (Liu et al., 2016). After normalization, significantly differentially expressed microRNAs between the two groups were identified using Fold change and *P*-value cutoffs of 2 and 0.05, respectively.

### Quantitative reverse-transcription polymerase chain reaction (qRT-PCR)

For qRT-PCR, a synthetic *Caenorhabditis elegans* microRNA, cel-miR-39 (Qiagen, Valencia, CA, USA), was added to plasma samples as a control prior to RNA extraction. Total RNA was extracted from 200 μL individual plasma samples with a miRNeasy extraction kit (Qiagen, Valencia, CA, USA) according to the manufacturer's instructions. RNA (6 μl) was reverse transcribed into cDNA (in a final volume of 10 μl) using a reverse transcription kit (GenePharma, Shanghai, China). Subsequently, quantitative real-time PCR was performed on an iQ™5 Real-Time PCR Detection System (Bio-Rid, USA) using SYBR Green. The primers used for qRT-PCR are listed in Table 1. Relative microRNA levels were determined by the 2^−ΔCT^ method.

**Table 1:** **Primers used for qRT-PCR verification of differently expressed circulating microRNAs**.

**microRNA**	**Forward primer**	**Reverse primer**
miR-369-3p	CAATGGAAATCGAATAATACATGG	TATGCTTGTTCTCGTCTCTGTGTC
miR-449b-3p	CGCGCCAGCCACAACTAC	TATGGTTGTTCACGACTCCTTCAC
miR-136-3p	CGGCGCATCATCGTCTCA	TATGGTTGTTCACGACTCCTTCAC
miR-4791	CGCGGCGCTGGATATGA	TATGGTTGTTCACGACTCCTTCAC
cel-miR-39	ATATCATCTCACCGGGTGTAAATC	TATGGTTTTGACGACTGTGTGAT

### Identifying potential biological relevance of microRNA signature

To explore the potential biological relevance of microRNAs signature, we predicted target genes of microRNAs using microT-CDS v5.0 (Paraskevopoulou et al., 2013) and TarBase v7.0 (Vlachos et al., 2015a). microT-CDS is an algorithm which is specifically trained on a positive and a negative set of microRNA recognition elements located in both the 3′-UTR and CDS regions, while TarBase is the largest available manually curated target database, indexing 9–250-fold more entries than any other available database. Both of them are associated with the latest miRBase version (v21) (Akhtar et al., 2016). Gene ontology (GO) enrichment analysis was performed for target genes using DIANA-miRPath v3. 0 which deciphers microRNA function with experimental support (Vlachos et al., 2015b). Network between microRNAs and target genes was constructed by Cytoscape v3.4.0 (Cline et al., 2007).

### Statistical analysis

Normality was assessed for all datasets by the Shapiro-Wilk's test. Then, independent *t*-test or Mann-Whitney *U*-test was carried out to assess baseline differences between AMS and non-AMS individuals. Comparison of the vitals mean difference between AMS and non-AMS groups using the two-way mixed ANOVA. Receiver operating characteristic curves were analyzed to assess specificity and sensitivity of single-plasma microRNAs and their combination using multiple logistic regression analysis. The optimal diagnostic point of the microRNAs was assessed at cut-off values with the largest Youden's index. To investigate the clinical impact of our signature microRNAs, we have chosen the likelihood ratio (LR) in addition to classical test parameters-sensitivity and specificity, because it combines information of both. Odds ratio per standard deviation was calculated by logistic regression analysis. Areas under the receiver-operating characteristic curves (AUCs) were evaluated using Swets classification (Gasparini et al., 2015): AUC = 0.5, no diagnostic value; 0.5 < AUC < 0.7, accuracy to only a small degree; 0.7 < AUC < 0.9, fair accuracy; 0.9 < AUC ≤ 1, high accuracy.

Statistical analyses were performed with the R software (version 3.2.3, R Foundation for Statistical Computing, Vienna, Austria) with package “OptimalCutpoints” (López-Ratón et al., 2014), IBM SPSS Statistics 19 (SPSS, Chicago, IL, USA, and GraphPad Prism 5. The following values were considered significant: *P* < 0.05(^*^) and *P* < 0.01(^**^).

## Results

### Clinical manifestation of all individuals

**Figure 1:**
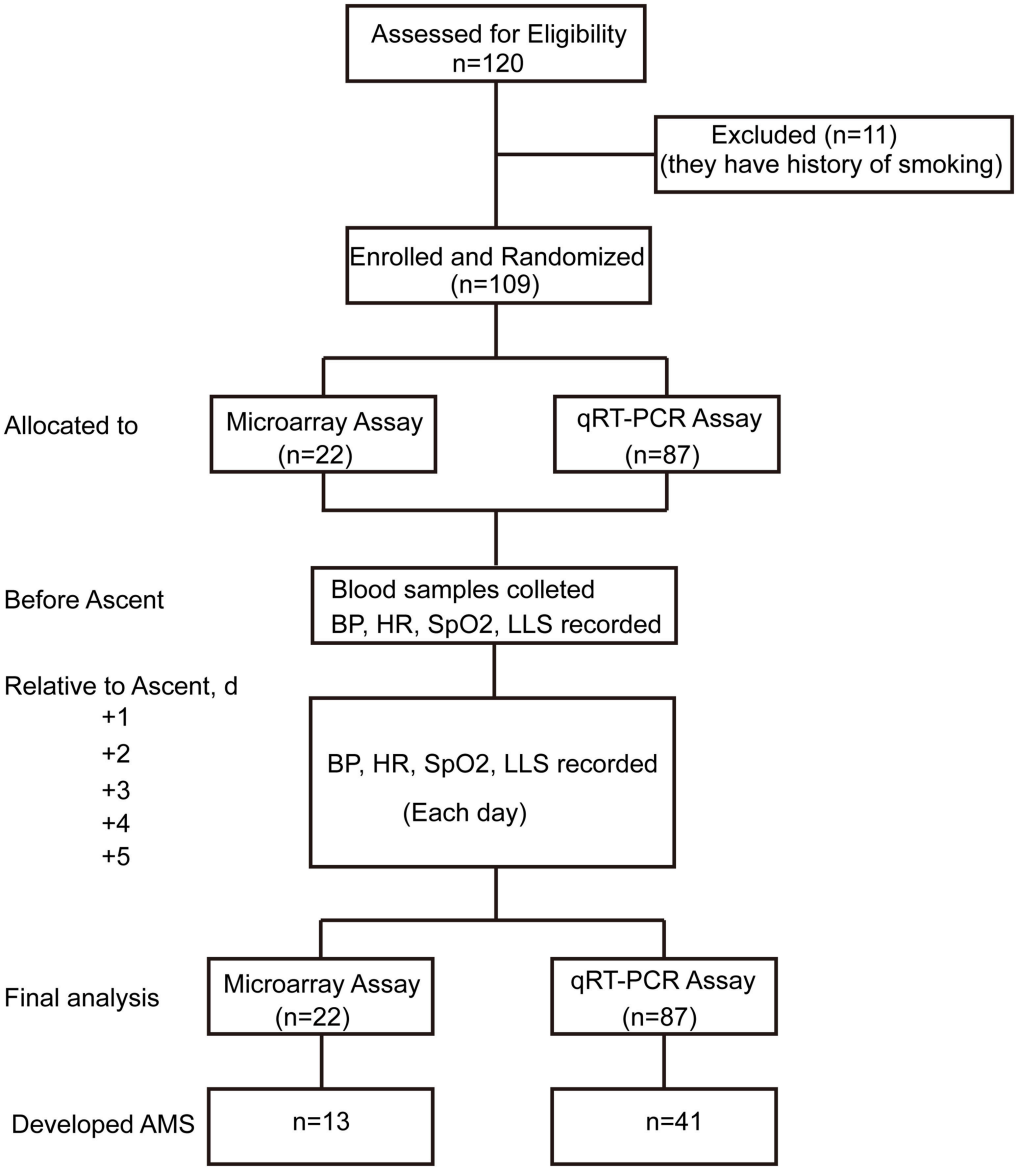
**Trial flow diagram**. AMS, acute mountain sickness; qRT-PCR, quantitative reverse-transcription polymerase chain reaction; LLS, Lake Louise Scoring System; BP, blood pressure; HR, heart rate; SpO2, oxygen saturation.

The trail flow diagram is shown in Figure 1. Assessed by LLS, 13 individuals were diagnosed as AMS and 9 as Non-AMS in microarray assay, while 41 volunteers were designated as AMS and 46 as Non-AMS in qRT-PCR test. In total, the morbidity of AMS is 49.5%. Respectively, there is no significant difference of age between AMS group and Non-AMS individuals in microRNA array screening set (23 ± 2.5 vs. 26 ± 6.5, *P* = 0.071) and qRT-PCR assay set (22 ± 3.5 vs. 22 ± 4.0, *P* = 0.081), as well as body mass index between AMS and Non-AMS groups (microRNA array screening set: 21 ± 2.5 vs. 22 ± 3.0, *P* = 0.475; qRT-PCR assay set: 22 ± 2.0 vs. 21 ± 4.0, *P* = 0.370; Table 2). After exposure to high altitude, oxygen saturation of all individuals significantly decreased, while heart rate significantly increased. However, only on the third day after exposure to high altitude, heart rate of AMS group was higher than Non-AMS individuals (Supplementary Table 2).

**Table 2:** **Characteristics of subjects**.

**Characteristics**	**AMS**	**Non-AMS**	***P*****-values**
**microRNAS ARRAY SCREENING SET (***n*** = 22)**			
**Age (Year)**			
Median ± IQR^*^	23 ± 2.5	26 ± 6.5	0.071
Range	24–32	24–35	
**Sex**			
Male	13	9	
Female	0	0	
BMI^*^ **(kg/m^2^)**			
Median ± IQR^*^	21 ± 2.5	22 ± 3.0	0.475
Range	20–25	19–26	
**qRT-PCR ASSAY SET (***n*** = 87)**			
**Age (Year)**			
Median ± IQR^*^	22 ± 3.5	22 ± 4.0	0.081
Range	17–27	17–26	
**Sex**			
Male	41	46	
Female	0	0	
BMI^*^ **(kg/m^2^)**			
Median ± IQR^*^	22 ± 2.0	21 ± 4.0	0.370
Range	18–25	18–26	

* *IQR, interquartile range; BMI, body mass index*.

### MicroRNA array and validation of microRNAs

**Figure 2:**
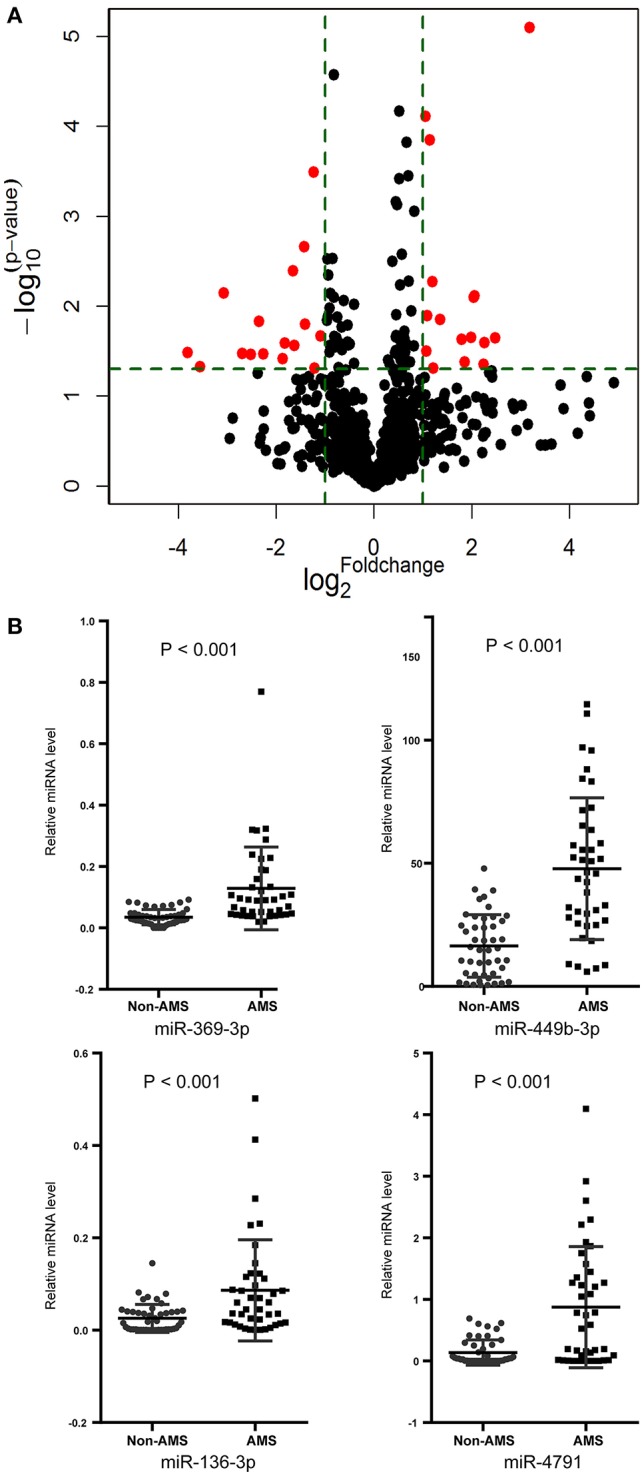
**Circulating microRNAs expression profile was different between acute mountain sickness (AMS) and non-acute mountain sickness (Non-AMS) groups. (A)** Comparisons of all microRNAs in microarray analysis of RNA isolated from plasma of AMS and Non-AMS groups. The volcano plot displays the relationship between fold-change and significance using a scatter plot view. The red points in the plot represent the differentially expressed microRNAs with statistical significance. **(B)** Results of the quantitative reverse-transcription polymerase chain reaction of the miR-369-3p, miR-449b-3p, miR-136-3p, and miR-4791 expression (AMS, *n* = 41; Non-AMS, *n* = 46).

MicroRNA array screening revealed that 31 microRNAs were differentially expressed between AMS and Non-AMS groups, 15 up-regulated and 16 down-regulated (Figure 2A). Among them, miR-369-3p, miR-449b-3p, miR-136-3p, and miR-4791 (MIMAT0019963) (all Fold changes > 5) were the most significantly up-regulated microRNAs in the AMS group compared with Non-AMS individuals. All the four microRNAs were considered for further validation with qRT-PCR assay.

Consistently, miR-369-3p, miR-449b-3p, miR-136-3p, and miR-4791 (all *P* < 0.001) were significantly up-regulated in AMS patients compared to Non-AMS individuals, using cel-miR-39 as normalization control, within qRT-PCR test (Figure 2B).

### MicroRNA signature for the identification of patients with acute mountain sickness

**Figure 3:**
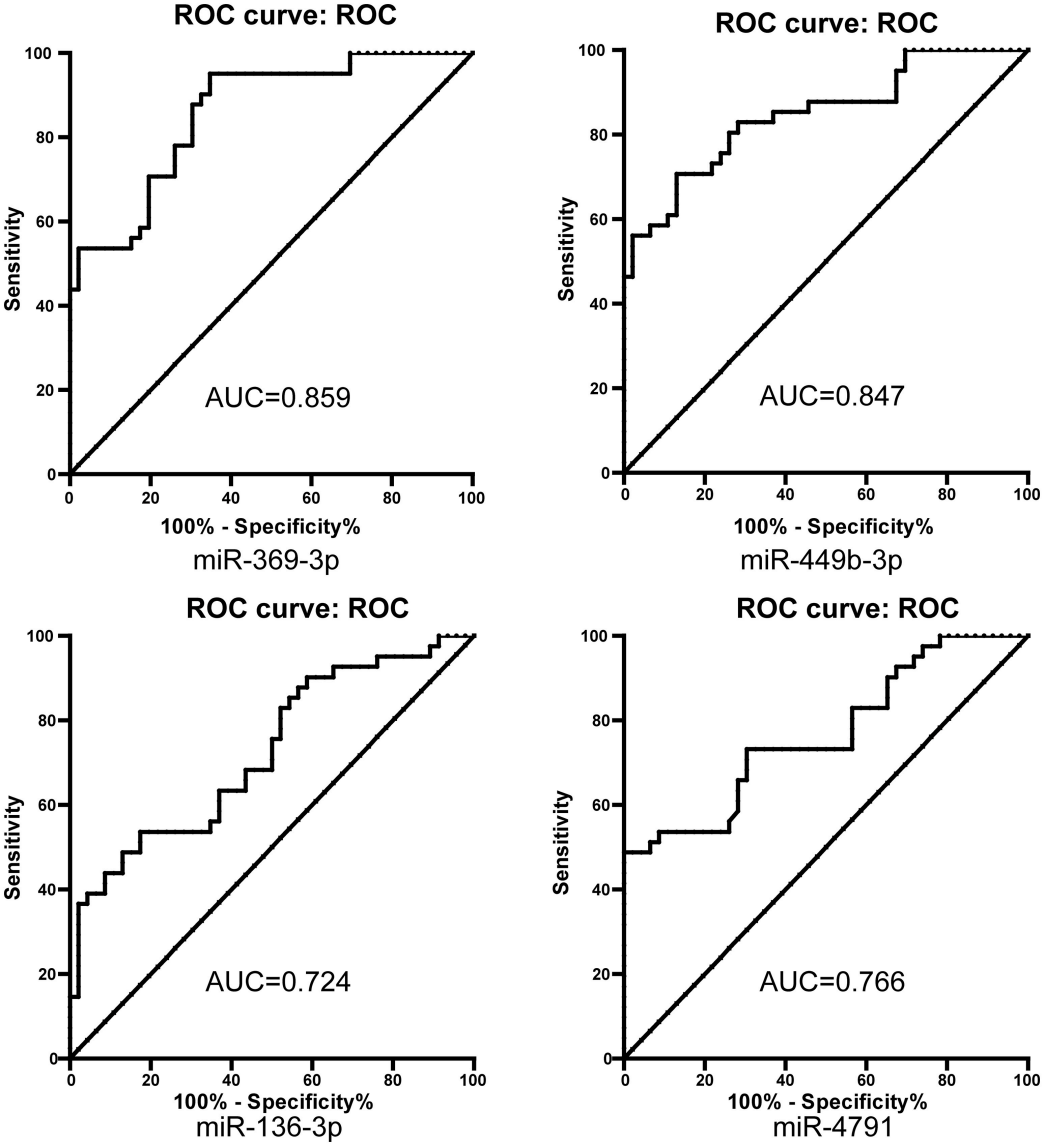
**ROC curve analysis for single microRNAs to discriminate acute mountain sickness patients from non-acute mountain sickness individuals**. AUC, area under curve.

The area under the curve (AUC) of miR-369-3p, miR-449b-3p, miR-136-3p, and miR-4791 was 0.859, 0.847, 0.724, and 0.766, respectively (Figure 3, Table 3). Among them, miR-369-3p present the highest accuracy for discrimination AMS from Non-AMS individuals. However, the AUC was <0.850 for the remind single-microRNA to predict AMS.

**Table 3:** **Receiver operating characteristic curves of single-plasma microRNAs**.

	**AUC**	**95% CI**	***P*****-values**	**OR per SD**	**95% CI**	**Cut off**	**Sensitivity (%)**	**95% CI**	**Specificity (%)**	**95% CI**	**LR+**	**LR−**
**AMS vs. Non-AMS**												
miR-369-3p	0.859	0.783–0.935	<0.001	62.586	8.032–487.699	>0.038	0.95	0.83–0.99	0.65	0.50–0.79	2.73	0.07
miR-449b-3p	0.847	0.765–0.929	<0.001	8.659	3.311–22.648	>29.602	0.71	0.54–0.84	0.87	0.74–0.95	5.42	0.33
miR-136-3p	0.724	0.617–0.831	<0.001	4.874	1.740–13.433	>0.045	0.54	0.37–0.69	0.83	0.69–0.92	3.08	0.56
miR-4791	0.766	0.666–0.867	<0.001	7.130	2.409–21.104	>0.091	0.73	0.57–0.86	0.70	0.54–0.82	2.40	0.39

*AUC, area under curve; CI, confidence interval; LR+, positive likelihood ratio; LR–, negative likelihood ratio; OR per SD, odds ratio per standard deviation; AMS, acute mountain sickness*.

Combination of markers could improve accuracy in disease diagnosis (Pepe and Thompson, 2000). So we performed receiver operating characteristic curves for combination of miR-369-3p, miR-449b-3p, miR-136-3p, and miR-4791 with Logistic Regression analysis. A binomial logistic regression was performed to ascertain the effects of miR-369-3p, miR-449b-3p, miR-136-3p, and miR-4791 on the likelihood that participants have AMS. Of the four predictor variables only three were statistically significant: miR-369-3p, miR-449b-3p, and miR-136-3p (Supplementary Table 3). Therefore, miR-4791 was excluded from the logistic regression model.

**Figure 4:**
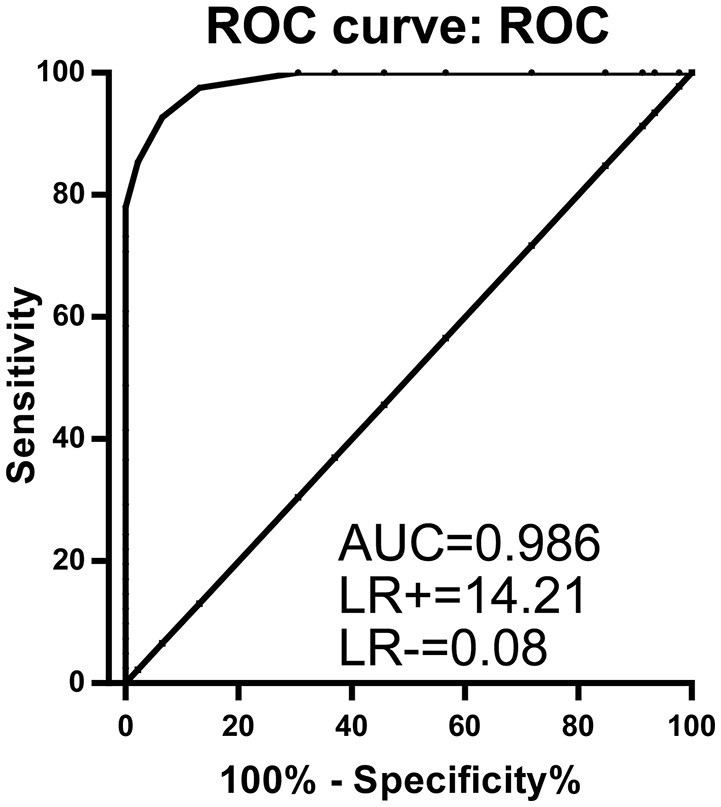
**ROC curve analysis for the combination of three microRNAs (miR-369-3p, miR-449b-3p, and miR-136-3p) resulting in enhanced specificity and sensitivity to distinguish acute mountain sickness patients from non-acute mountain sickness individuals (97.6, 93.4%, respectively)**. AUC, area under curve; LR, likelihood ratio.

The logistic regression model for miR-369-3p, miR-449b-3p, and miR-136-3p was statistically significant, $\chi_{\left(3\right)}^{2}$ = 95.616, *P* < 0.001. The model explained 89.0% (Nagelkerke *R*^2^) of the variance in AMS and correctly classified 94.3% of cases. All predictor variables were statistically significant (Supplementary Table 4). Notably, the combination of miR-369-3p, miR-449b-3p, and miR-136-3p resulted in a robustly increased AUC (0.986, 95%CI 0.970–1.000; LR+: 14.21; LR−: 0.08), leading to a signature for the prediction of AMS (Figure 4). Accordingly, despite the single-microRNA cut-off values between AMS and Non-AMS (>0.038 for miR-369-3p, >29.602 for miR-449b-3p, and >0.045 for miR-136-3p; Table 3), a global cut-off value of >0.110 for the signature of miR-369-3p, miR-449b-3p, and miR-136-3p combination increased the diagnostic power to a 92.68% sensitivity and a 93.48% specificity.

### Biological relevance of microRNA signature

**Figure 5:**
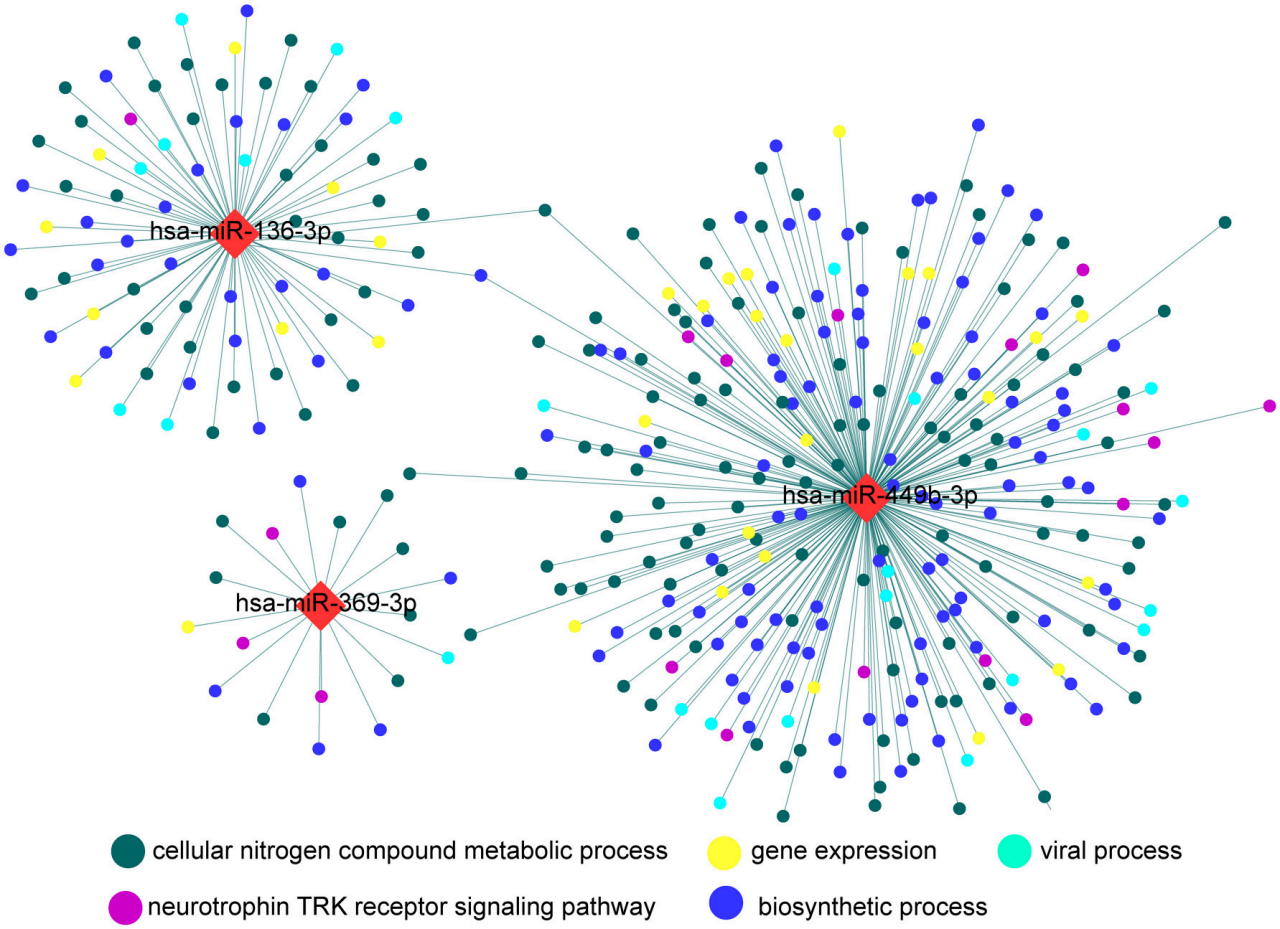
**Direct interaction network between microRNAs and target genes**. The diamond nodes represent microRNAs, while circular nodes represent target genes. Color of circular nodes represents the most enriched association with a top five GO biological process. GO, Gene ontology.

GO enrichment analysis for target genes of microRNA signature revealed that cellular nitrogen compound metabolic process, gene expression, neurotrophin TRK receptor signaling pathway, biosynthetic process, and viral process were the top five GO term over-represented in target genes of the microRNA signature (Figure 5, Supplementary Table 5).

## Discussion

The number of individuals who ascended to high altitude for a variety of reasons is increasing. AMS which can progress to high altitude cerebral edema is a common problem to them (Honigman et al., 1993; Hackett and Roach, 2001; Karinen et al., 2008). Speed of ascent (Hackett et al., 1976), previous history of AMS (Schneider et al., 2002), obesity (Ri-Li et al., 2003), migraine (Mairer et al., 2009), and so forth (Taylor, 2011) have been identified as risk factors for AMS. However, the incidence of AMS is different in individuals, families and populations because of their different capacity to cope with high-altitude environments (MacInnis et al., 2010). This study for the first time reports a novel signature of three circulating microRNAs allowing to distinguishing AMS from Non-AMS individuals before exposure to high altitude.

Nowadays, there are many ways to prevent AMS through non-pharmacologic and pharmacologic measures (Imray et al., 2010). Providing time for the body to acclimatize to high altitude is the single best way to prevent AMS (Beidleman et al., 2004; Luks et al., 2014), but it is not impracticable to individuals with urgent task. Pharmacologic prophylaxis is an appropriate way for the majority of individuals; however, the adverse effect of pharmaceutical products could impair the health of people (Dumont et al., 2000). Thus, identifying individuals susceptible to AMS would assist in the development of suitable preventive strategies for a given individual.

The study here provided a more effective method to detect individuals at risk of AMS with circulating microRNAs than those used currently. The receiver operating characteristic curves revealed that the power of diagnostic test for AMS with a 92.68% sensitivity and a 93.48% specificity. Although already one microRNA could discriminate to AUC of 0.859 between the different groups, this approach failed in appropriate LR. Only the combination of three microRNAs managed to deliver an increased discrimination (AUC 0.986) with a high positive LR in accordance with a low negative LR.

The biological relevance of circulating microRNAs is now regarded as a global, hormone-like functional molecule that might allow regulation of gene expression across tissues at a distance (Turchinovich et al., 2013). Here, we found that the expression value of microRNA signature in AMS susceptible individuals was significantly higher than AMS resistant individuals. Its target genes more enriched in cellular nitrogen compound metabolic process and neurotrophin TRK receptor signaling pathway. Nitric oxide (NO), generated through cellular nitrogen compound metabolic process, is a major signaling and effector molecule mediating the body's response to hypoxia (Beall et al., 2012). The generation of high levels of NO and circulating nitrogen oxide species promote the adaptation of natives of Tibet and acclimatization of lowlanders who exposure to high altitude via enabling greater blood flow and oxygen delivery (Erzurum et al., 2007; Janocha et al., 2011). Neurotrophin TRK receptor is high-affinity receptor of brain-derived neurotrophic factor whose concentration will be increased in human plasma after exposure to high altitude (elevation: 3350 m) for 72 h (Helan et al., 2014). Prakash and his colleagues confirmed that brain-derived neurotrophic factor could induce NO generation in human pulmonary artery endothelial cells via TRK receptor (Meuchel et al., 2011).

Previously studies affirm that high levels of NO are associated with function benefits and the avoidance of illness (Janocha et al., 2011). The higher expression value of microRNA signature in AMS susceptible individuals will suppress the genes involved in cellular nitrogen compound metabolic process and neurotrophin TRK receptor signaling pathway, post-transcriptionally and cause a low levels of NO and circulating nitrogen oxide species. Thus, the blunt production of NO and circulating nitrogen oxide species via microRNAs may be the biological underpinning of AMS susceptible individuals who present a higher expression level of the microRNA signature consisted of miR-369-3p, miR-449b-3p, and miR-136-3p.

We demonstrated for the first time a unique signature of circulating microRNAs to differentiate AMS from Non-AMS individuals. However, these results need to be confirmed in a large patient cohort to exclude potential bias. And only the young health men were included in this study because they are the main part of population who travel to high altitudes for recreation, work, and pilgrimage. Therefore, further investigations in more individuals, also including females, other races and ages, should be obtained to affirm the performance of the signature proposed here.

## Conclusion

Here, we, for the first time, describe a signature of circulating microRNAs for sensitive and specific identification of the susceptibility of AMS before exposure to high altitude. This signature may hold great promise to become an important tool for the prediction of AMS.

## Availability of data and materials

All data have been submitted to GEO under the accession GSE90500.

## Author contributions

YG conceived and designed the study. HH and JC oversaw laboratory analyses and BL provided the overall supervision of the study. GW, EZ, and GX did the laboratory experiments or contributed the statistical analysis, or both GW and BS contributed to sample and physical data collections. BL drafted the report. All authors contributed to the interpretation of results, critical revision of the manuscript, and approved the final manuscript. YG is the guarantor.

### Conflict of interest statement

The authors declare that the research was conducted in the absence of any commercial or financial relationships that could be construed as a potential conflict of interest.

## Abbreviations

AMS: acute mountain sickness
qRT-PCR: quantitative reverse-transcription polymerase chain reaction
LLS: Lake Louise Scoring System
AUC: area under the receiver-operating characteristic curve
LR: likelihood ratio
NO: nitric oxide
GO: gene ontology.
