# Supplementary material for: A Signature of Circulating microRNAs Predicts the Susceptibility of Acute Mountain Sickness
Source: Front Physiol. 2017 Feb 8;8:55. doi: 10.3389/fphys.2017.00055 (PMC5296306; doi:10.3389/fphys.2017.00055)
Supplement: Supplementary file 1 [file Table1.DOCX]

Supplementary Table 1 RNA Quantification and Quality Assurance by NanoDrop ND-1000

| SampleID | OD260/280 Ratio | OD260/230 Ratio | Conc.(ng/μl) | Volume(μl) | Quantity(ng) |
| --- | --- | --- | --- | --- | --- |
| P1 | 1.70 | 0.65 | 40.29 | 10 | 402.9 |
| P2 | 1.46 | 0.73 | 33.20 | 10 | 332.0 |
| P3 | 1.70 | 0.65 | 30.41 | 10 | 304.1 |
| P4 | 1.46 | 0.73 | 34.04 | 10 | 340.4 |
| P5 | 1.70 | 0.65 | 29.89 | 10 | 298.9 |
| P6 | 1.46 | 0.73 | 31.05 | 10 | 310.5 |
| P7 | 1.70 | 0.65 | 31.42 | 10 | 314.2 |
| P8 | 1.46 | 0.73 | 28.52 | 10 | 285.2 |
| P9 | 1.70 | 0.65 | 30.42 | 10 | 304.2 |
| P10 | 1.46 | 0.73 | 26.92 | 10 | 269.2 |
| P11 | 1.70 | 0.65 | 26.96 | 10 | 269.6 |
| P12 | 1.46 | 0.73 | 28.65 | 10 | 286.5 |
| P13 | 1.70 | 0.65 | 21.80 | 10 | 218.0 |
| P14 | 1.46 | 0.73 | 24.84 | 10 | 248.4 |
| P15 | 1.70 | 0.65 | 24.39 | 10 | 243.9 |
| P16 | 1.46 | 0.73 | 21.74 | 10 | 217.4 |
| P17 | 1.70 | 0.65 | 49.68 | 10 | 496.8 |
| P18 | 1.46 | 0.73 | 20.39 | 10 | 203.9 |
| P19 | 1.70 | 0.65 | 29.56 | 10 | 295.6 |
| P20 | 1.46 | 0.73 | 29.02 | 10 | 290.2 |
| P21 | 1.70 | 0.65 | 28.77 | 10 | 287.7 |
| P22 | 1.46 | 0.73 | 27.15 | 10 | 271.5 |
