# Supplementary material for: A Signature of Circulating microRNAs Predicts the Susceptibility of Acute Mountain Sickness
Source: Front Physiol. 2017 Feb 8;8:55. doi: 10.3389/fphys.2017.00055 (PMC5296306; doi:10.3389/fphys.2017.00055)
Supplement: Supplementary file 2 [file Table2.DOCX]

Supplementary Table 2 Basic physiological data^#^ based on 54 acute mountain sickness (AMS) patients and 55 Non-acute mountain sickness (Non-AMS) individuals. ^#^Data are presented as mean (SD) or median (IQR). **P*<0.05, ***P*<0.01 compared with Non-AMS

| Variables | Group | Plain | High Altitude | | | | |
| --- | --- | --- | --- | --- | --- | --- | --- |
|  |  |  | Day1 | Day2 | Day3 | Day4 | Day5 |
| Altitude, m |  | 200 | 3648 | 3648 | 3648 | 3648 | 3648 |
| Oxygen saturation,% | AMS | 97.4(0.8) | 84.5(4) | 85(4.4) | 85.5(4.3) | 86.2(4.7) | 87.7(3.8) |
|  | Non-AMS | 97.7(0.7) | 85.7(3.6) | 86.1(4.3) | 87.1(3) | 86(4.3) | 87.5(3.5) |
| Heart rate, bpm | AMS | 74(11) | 86(11) | 90(13) | 94(12)^**^ | 93(13) | 88(13) |
|  | Non-AMS | 74(10) | 88(12) | 91(13) | 87(14) | 89(12) | 87(12) |
| Systolic blood pressure, mmHg | AMS | 116(10) | 118(11) | 122(9) | 123(9) | 123(11) | 123(12) |
|  | Non-AMS | 119(11) | 121(12) | 121(12) | 121(10) | 122(12) | 120(11) |
| Diastolic blood pressure, mmHg | AMS | 68(8) | 72(10) | 74(11)^*^ | 72(10) | 77(10)^**^ | 76(11)^*^ |
|  | Non-AMS | 68(8) | 69(11) | 69(10) | 71(10) | 71(10) | 70(10) |
| Lake Louise score | AMS | 0(0) | 4(3)^**^ | 5(2)^**^ | 4(4)^**^ | 3(4)^**^ | 2(4)^**^ |
|  | Non-AMS | 0(0) | 0(1) | 0(1) | 0(1) | 0(0) | 0(0) |
