# Supplementary material for: A Signature of Circulating microRNAs Predicts the Susceptibility of Acute Mountain Sickness
Source: Front Physiol. 2017 Feb 8;8:55. doi: 10.3389/fphys.2017.00055 (PMC5296306; doi:10.3389/fphys.2017.00055)
Supplement: Supplementary file 3 [file Table3.docx]

Supplementary Table 3 Logistic Regression Predicting Likelihood of Acute Mountain Sickness base on miR-369-3p, miR-449b-3p, miR-136-3p and miR-4791

|  | B | SE | Wald | df | p | Odds Ratio | 95% CI for Odds Ratio | |
| --- | --- | --- | --- | --- | --- | --- | --- | --- |
|  |  |  |  |  |  |  | Lower | Upper |
| miR-369-3p | 130.72 | 49.10 | 7.09 | 1 | 0.008 | 5.92E+56 | 9.59E+14 | 3.65E+98 |
| miR-449b-3p | 0.24 | 0.12 | 4.13 | 1 | 0.042 | 1.26 | 1.01 | 1.59 |
| miR-136-3p | 45.70 | 20.35 | 5.04 | 1 | 0.025 | 7.04E+19 | 336.67 | 1.47E+37 |
| miR-4791 | 5.17 | 3.08 | 2.82 | 1 | 0.093 | 176.55 | 0.42 | 74030.55 |
| Constant | -17.89 | 6.80 | 6.93 | 1 | 0.008 | 121.25 |  |  |
