# Supplementary material for: A Signature of Circulating microRNAs Predicts the Susceptibility of Acute Mountain Sickness
Source: Front Physiol. 2017 Feb 8;8:55. doi: 10.3389/fphys.2017.00055 (PMC5296306; doi:10.3389/fphys.2017.00055)
Supplement: Supplementary file 4 [file Table4.docx]

Supplementary Table 4 Logistic Regression Predicting Likelihood of Acute Mountain Sickness base on miR-369-3p, miR-449b-3p and miR-136-3p

|  | B | SE | Wald | df | p | Odds Ratio | 95% CI for Odds Ratio | |
| --- | --- | --- | --- | --- | --- | --- | --- | --- |
|  |  |  |  |  |  |  | Lower | Upper |
| miR-369-3p | 133.90 | 46.32 | 8.36 | 1 | 0.004 | 1.43E+58 | 5.35E+18 | 3.79E+97 |
| miR-449b-3p | 0.18 | 0.06 | 8.22 | 1 | 0.004 | 1.20 | 1.06 | 1.36 |
| miR-136-3p | 33.94 | 15.00 | 5.13 | 1 | 0.024 | 5.49E+14 | 95.23 | 3.16E+27 |
