# Supplementary material for: A Signature of Circulating microRNAs Predicts the Susceptibility of Acute Mountain Sickness
Source: Front Physiol. 2017 Feb 8;8:55. doi: 10.3389/fphys.2017.00055 (PMC5296306; doi:10.3389/fphys.2017.00055)
Supplement: Supplementary file 5 [file Table5.docx]

Supplementary table 5 Gene ontology (biological process) enrichment analysis for target genes of microRNA signature consisted of has-miR-369-3p, has-miR-449b-3p and has-miR-136-3p.

| **GO_id** | **GO Term** | **p-value** | **FDR** | **#genes** | **#miRNAs** |
| --- | --- | --- | --- | --- | --- |
| GO:0034641 | cellular nitrogen compound metabolic process | 2.13E-13 | 4.62E-10 | 154 | 3 |
| GO:0010467 | gene expression | 1.27E-11 | 1.38E-08 | 33 | 3 |
| GO:0048011 | neurotrophin TRK receptor signaling pathway | 2.88E-09 | 2.08E-06 | 18 | 3 |
| GO:0016032 | viral process | 1.81E-08 | 7.84E-06 | 25 | 3 |
| GO:0009058 | biosynthetic process | 1.73E-08 | 7.84E-06 | 124 | 3 |
| GO:0044403 | symbiosis, encompassing mutualism through parasitism | 8.29E-08 | 3.00E-05 | 26 | 3 |
| GO:0030168 | platelet activation | 3.62E-07 | 0.000112123 | 15 | 3 |
| GO:0006464 | cellular protein modification process | 4.28E-07 | 0.000116098 | 77 | 3 |
| GO:0008219 | cell death | 6.63E-07 | 0.00015987 | 40 | 3 |
| GO:0007173 | epidermal growth factor receptor signaling pathway | 7.88E-07 | 0.000170983 | 15 | 3 |
| GO:0044267 | cellular protein metabolic process | 3.68E-06 | 0.000686684 | 21 | 3 |
| GO:0007268 | synaptic transmission | 4.12E-06 | 0.000686684 | 22 | 3 |
| GO:0008150 | biological_process | 3.89E-06 | 0.000686684 | 453 | 3 |
| GO:0043687 | post-translational protein modification | 8.94E-06 | 0.001385612 | 11 | 3 |
| GO:0061024 | membrane organization | 2.16E-05 | 0.003117882 | 25 | 3 |
| GO:0016071 | mRNA metabolic process | 3.83E-05 | 0.004890161 | 12 | 2 |
| GO:0007267 | cell-cell signaling | 3.78E-05 | 0.004890161 | 28 | 3 |
| GO:0018279 | protein N-linked glycosylation via asparagine | 7.73E-05 | 0.008825339 | 8 | 3 |
| GO:0007596 | blood coagulation | 7.41E-05 | 0.008825339 | 19 | 3 |
| GO:0009056 | catabolic process | 9.40E-05 | 0.010197568 | 60 | 3 |
| GO:0007220 | Notch receptor processing | 0.000177855 | 0.018369862 | 5 | 2 |
| GO:0016192 | vesicle-mediated transport | 0.000202058 | 0.019921058 | 39 | 3 |
| GO:0044281 | small molecule metabolic process | 0.000225515 | 0.021267036 | 67 | 3 |
| GO:0002576 | platelet degranulation | 0.00029364 | 0.025547824 | 6 | 2 |
| GO:0008543 | fibroblast growth factor receptor signaling pathway | 0.000294465 | 0.025547824 | 11 | 3 |
| GO:0038095 | Fc-epsilon receptor signaling pathway | 0.000451768 | 0.037687874 | 8 | 2 |
